# High-Fidelity Transcriptome Reconstruction of Degraded RNA-Seq Samples Using Denoising Diffusion Models

**DOI:** 10.3390/biology14121652

**Published:** 2025-11-23

**Authors:** Ke Xiao, Jinlei Sun, Yunqing Liu, Chen Li, Hengchuan Guo, Yiying Wang, Yajun Pang, Zhiyu Liu

**Affiliations:** 1 State Key Laboratory of Digital Medical Engineering, School of Biological Science and Medical Engineering, Southeast University, Nanjing 211189, China; kexiao@seu.edu.cn; 2 School of Computer Science, Luoyang Institute of Science and Technology, Luoyang 471000, China; jlsun@lit.edu.cn (J.S.); liuyq@lit.edu.cn (Y.L.); ieliubl@lit.edu.cn (C.L.); guohengchuan@lit.edu.cn (H.G.); 17605985265@163.com (Y.W.); 3 Office of Academic Research, Nanjing Normal University of Special Education, Nanjing 210038, China

**Keywords:** RNA sequencing, RNA degradation, transcriptome repair, diffusion model, Transformer, bioinformatics, deep learning

## Abstract

Background: RNA degradation in clinically archived samples introduces systematic biases into RNA-seq data, limiting the accuracy of downstream analyses. Developing computational methods for high-fidelity transcriptome restoration is therefore of critical importance. Methods: We introduce DiffRepairer, a deep learning model that combines a Transformer architecture with a conditional diffusion model framework to reverse the effects of RNA degradation. The model is trained on “degraded-original” paired data, generated via a comprehensive simulation pipeline, to learn a direct, one-step repair mapping. Results: Across five diverse pseudo-degraded datasets, DiffRepairer demonstrated stable and superior performance, outperforming traditional statistical methods (e.g., CQN) and standard deep learning models (e.g., VAE) on key technical and biological metrics. Conclusion: DiffRepairer is a validated, high-precision tool for transcriptome repair that effectively restores biologically meaningful signals from degraded RNA-seq data, highlighting the potential of advanced generative models in bioinformatics.

## Simple Summary

RNA degradation is a major technical barrier to genomic research using clinically archived samples, as it distorts gene expression data and hinders scientific discovery. To address this challenge, we developed DiffRepairer, a deep learning framework that combines the Transformer architecture with concepts from diffusion models. By learning the inverse mapping of the degradation process, the framework aims to computationally reconstruct the original transcriptome with high fidelity. A comprehensive evaluation across five diverse datasets shows that DiffRepairer systematically outperforms existing statistical and deep learning methods in both reconstruction accuracy and the preservation of key biological signals, such as differentially expressed genes. DiffRepairer provides a validated computational tool for unlocking the scientific value of vast archives of biological samples, opening new possibilities for discovering reliable disease biomarkers and therapeutic targets from low-quality data.

## 1. Introduction

The quality of transcriptomic data is a prerequisite for obtaining reliable biological insights. RNA molecules are inherently unstable, and within living cells, their quality is controlled by a sophisticated network of post-transcriptional regulation. However, once a sample is ex vivo, particularly during the preparation and storage of formalin-fixed paraffin-embedded (FFPE) samples commonly used in clinical research [1], RNA is highly susceptible to degradation by ribonucleases (RNases) and chemical modifications. These samples, numbering in the hundreds of millions in pathology archives worldwide, are an invaluable resource for retrospective clinical studies, but their nucleic acid quality poses a major technical bottleneck for genomic analysis. This degradation process typically manifests as random fragmentation of RNA strands. Because standard RNA-seq library preparation protocols (e.g., poly-A enrichment) rely on intact 3′ ends, RNA fragmentation disproportionately leads to the loss of 5′ transcript information, creating a 3′ bias in the sequencing data. As degradation intensifies, low-abundance transcripts may be completely fragmented or lost, leading to gene signal dropout. Furthermore, sample processing and sequencing introduce random technical noise. These factors combine to distort the original gene expression profile of a sample, challenging downstream analyses such as cancer subtyping, biomarker discovery, and drug response prediction. Recent experimental studies have shown that a mere 24 h delay in sample processing is sufficient to cause significant distortion of the transcriptome profile (gene expression correlation dropping below 0.8) and to systematically activate cellular stress response pathways while suppressing key immune function pathways, thereby severely compromising the reliability of downstream analysis [2]. Consequently, developing tools that can effectively correct these biases and computationally repair and restore the original transcriptome from low-quality data has become a pressing scientific problem.

High-quality RNA-seq reference atlases, such as those from the Gene Expression Omnibus (GEO) [3], provide an ideal “gold standard” depicting gene expression patterns in an undegraded state. This offers a viable path for computational repair: building a model that learns the mapping from degraded data to its corresponding high-quality counterpart. However, many existing computational methods, such as MAGIC [4] or Scaden for single-cell data imputation, were originally designed to address sparsity due to low capture efficiency and do not comprehensively account for the complex, co-existing patterns of biases unique to RNA degradation. Moreover, many traditional methods rely on linear assumptions or specific statistical distributions, limiting their expressive power when faced with complex, non-linear gene regulatory networks, and thus struggle to achieve high-fidelity repair.

To overcome these limitations, we turned to deep generative models. Denoising Diffusion Probabilistic Models (DDPMs) [5], which have recently achieved breakthroughs in fields like image generation, offer a new perspective. In recent years, foundation and generative models for single-cell omics have rapidly advanced and been applied to data generation, imputation, and multi-omics integration [6,7,8]. We analogize the biological degradation of RNA—a process where molecules become progressively disordered and entropy increases—to the forward diffusion process of a diffusion model. Within this conceptual framework, the task of transcriptome repair can be viewed as learning the reverse process. However, unlike traditional DDPMs that require an iterative multi-step sampling process, our approach is optimized for the computational demands of large-scale omics data. We model the repair as a single-step denoising map, where the model directly learns the end-to-end mapping from a degraded state to its original state. This can be conceptualized as learning the terminal distribution of a diffusion process, akin to the one-step generation idea in recent Consistency Models [9], maximizing efficiency without sacrificing the generative framework’s conceptual power.

In this paper, we propose DiffRepairer, a transcriptome repair framework based on the concept of conditional diffusion models and the Transformer architecture [10]. We chose the Transformer as the core network because transcriptome repair requires understanding the complex interdependencies among thousands of genes. The self-attention mechanism of the Transformer can capture global dependencies in the gene expression profile, enabling more accurate and context-aware judgments during the repair process. Our systematic evaluation follows a comprehensive strategy: first, we validated our method on five pseudo-degraded datasets covering different tissue types; second, we compared its performance against several established baseline methods, including traditional statistical approaches and standard deep learning models, to thoroughly assess its effectiveness.

## 2. Materials and Methods

### 2.1. Datasets and Preprocessing

This study aims to develop a computational model capable of repairing low-quality RNA-seq data. Due to the extreme difficulty of experimentally obtaining “high-quality/low-quality” paired data from the same biological sample for model training, we adopted a standard strategy in the field: starting from publicly available high-quality datasets and generating pseudo-degraded paired samples for training through computational simulation.

To this end, we selected five high-quality single-cell or bulk RNA-seq datasets as the ground truth. These datasets cover a diverse range of biological samples, from the immune system to solid organs, providing a solid foundation for the model to learn true transcriptome structures.

- PBMC3k and PBMC68k Datasets: These two datasets from 10x Genomics (Pleasanton, CA, United States) [11] contain single-cell expression profiles of peripheral blood mononuclear cells (PBMCs) from healthy human donors.
- Brain Dataset: This dataset, from a single-cell RNA-seq study of the mouse brain [12], provides the complex cellular heterogeneity of the nervous system.
- Liver Dataset: This dataset from a study on the human liver offers high-quality transcriptome data of liver tissue [13].
- Pancreas Dataset: This dataset from a study on human pancreatic tissue features a complex cellular composition [14].

To ensure a fair comparison of methods, we applied a uniform preprocessing pipeline to all datasets, which was consistent across all compared methods (DiffRepairer, DAE, VAE, CQN, MAGIC):

1. Quality Control (QC): We filtered out cells with too few or too many expressed genes, as well as cells with a high percentage of mitochondrial genes. Genes expressed in very few cells were also removed [15].
2. Normalization and Transformation: Gene expression counts were normalized to counts per 10,000 (CP10k) and then log-transformed (log1p) [16].
3. Feature Selection: For each dataset, we selected the top 5000 highly variable genes (HVGs) as input features for the model.

### 2.2. Pseudo-Degradation Data Simulation

To train and evaluate our repair model, we generated pseudo-degraded samples with known “degraded-original” correspondences from the preprocessed high-quality data. While RNA degradation at the molecular level is influenced by factors such as nucleotide context, our simulation aims to model the well-documented, macro-level technical artifacts that corrupt the overall expression profile. These include systematic biases like 3′ transcript bias and stochastic events like gene signal dropout. More importantly, factors such as processing delays can induce systematic stress responses in cells, leading to the upregulation of specific biological pathways (e.g., inflammatory response) and the downregulation of functional pathways (e.g., interferon response). Therefore, our simulation process aims to comprehensively replicate these complex degradation patterns, which are composed of both random physical degradation and systematic biological stress. This process is consistent with the core ideas of established methods in single-cell transcriptome simulation (e.g., Splatter [17]) and correction strategies for degraded samples, and can be summarized by the following formula:

(1) $$X_{deg}=\left(M⊙X_{orig}\right)⊙d+\epsilon$$

where the degradation process consists of three independent modules:

1. 3′ Bias: This effect is one of the most typical biases in archived samples like FFPE. It is modeled by a bias matrix *M*, which simulates the loss of 5′ transcript signal due to RNA fragmentation. Each element of the matrix *M* is calculated based on the degradation intensity parameter α and gene length, disproportionately reducing the expression signals of longer transcripts.
2. Gene Dropout: This is a common artifact in low-quality sequencing data, especially in single-cell data. It is modeled by a binary mask vector m, where each element $m_{g}∼Bernoulli\left(1-p_{d}\right)$, with $p_{d}$ being the dropout rate. The mask is applied through element-wise multiplication with the expression vector, setting some gene expression values to zero with a certain probability.
3. Technical Noise: This is simulated by additive Gaussian noise ϵ, where $\epsilon ∼N\left(0,\sigma_{n}^{2}I\right)$, and $\sigma_{n}$ is the noise standard deviation. This component reproduces the random fluctuations introduced during sequencing and other technical steps, a standard practice in many data simulators.

By adjusting the parameters α, $p_{d}$, and$\;\sigma_{n}$, we can generate degradation data with different patterns and intensities, which are then partitioned into training, validation, and test sets for subsequent model training and evaluation.

### 2.3. Orthogonal Validation of Degradation Simulation

As obtaining true paired degraded samples (i.e., a high-quality version and a naturally degraded version from the same biological specimen) is experimentally challenging, this study relies on computationally simulated degradation data. To verify that our degradation simulation strategy can realistically replicate the impact of RNA degradation on biological signals, we used CITE-seq (Cellular Indexing of Transcriptomes and Epitopes by Sequencing) [18] for orthogonal validation.

Validation Strategy: CITE-seq allows for the simultaneous measurement of RNA transcript expression and surface protein expression at the single-cell level. This provides an independent biological measurement dimension: if our degradation simulation is realistic, the consistency between the simulated degraded RNA data and the protein data (which is unaffected by our simulation) should significantly decrease.

Experimental Design: We used a publicly available PBMC CITE-seq dataset from 10× Genomics (approx. 10,000 cells), containing both RNA-seq data and expression data for 17 surface protein markers. We selected 10,000 highly variable genes for analysis and successfully matched 6 representative protein-gene pairs, including key markers for immune cells: CD3 (CD3E), CD4 (CD4), CD8 (CD8A), CD14 (CD14), CD16 (FCGR3A), and CD127 (IL7R).

Evaluation Method: For each protein–gene pair, we calculated the Spearman correlation coefficient between RNA expression and protein expression and compared it under two data conditions: true high-quality RNA data vs. protein data; and simulated degraded RNA data vs. protein data (protein data remained unchanged).

Validation Results: The logic of this orthogonal validation is to first demonstrate that our simulation disrupts biological consistency, and then—critically—to show that our model can restore it. In the true data, the average RNA-protein correlation was 0.575 ± 0.168. After our degradation simulation was applied to the RNA data, this correlation dropped to 0.134 ± 0.068.

**Figure A1:**
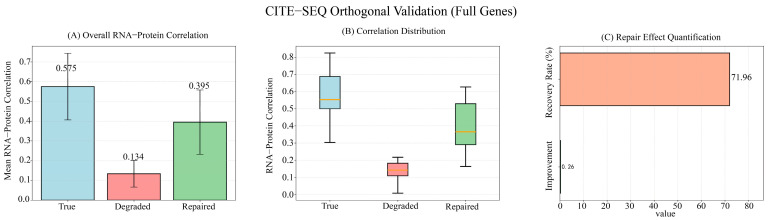
Complete results of the CITE-seq orthogonal validation. (**A**) Comparison of RNA-protein correlations in true, degraded, and repaired data. Degradation caused the correlation to drop from 0.575 to 0.134 (a 77% decrease), demonstrating the realism of the degradation simulation. After repair with DiffRepairer, the correlation recovered to 0.395 (a 72.0% recovery rate), proving the model’s effectiveness. (**B**) Box plot of correlation distributions, showing the statistical significance of the repair effect (*p* = 0.003). (**C**) Quantification of the repair effect: a 0.262 improvement in correlation and a 72.0% recovery rate. This orthogonal validation, using independent protein expression data, provides biological evidence for both the realism of the degradation simulation and the effectiveness of the model repair.

While any data alteration can reduce correlation, the crucial evidence lies in the subsequent recovery: after applying DiffRepairer to the degraded data, the RNA-protein correlation recovered to 0.395 ± 0.163, representing a 72.0% recovery of the lost correlation (paired *t*-test, *p* = 0.003). This recovery is the key validation. If our degradation simulation were merely introducing random noise, it would be impossible for any model to systematically recover the biological signal. The fact that DiffRepairer can restore RNA-protein correlation provides strong, independent evidence that: (1) our simulation introduces realistic, structured technical artifacts (not just random deletion), and (2) our model has learned genuine biological relationships that generalize to an independent measurement modality never used in training. This two-step validation (disruption then recovery) confirms both the biological relevance of our simulation and the mechanistic efficacy of our model (see Figure A1 for detailed results).

### 2.4. DiffRepairer Model Architecture and Training

**Figure 1:**
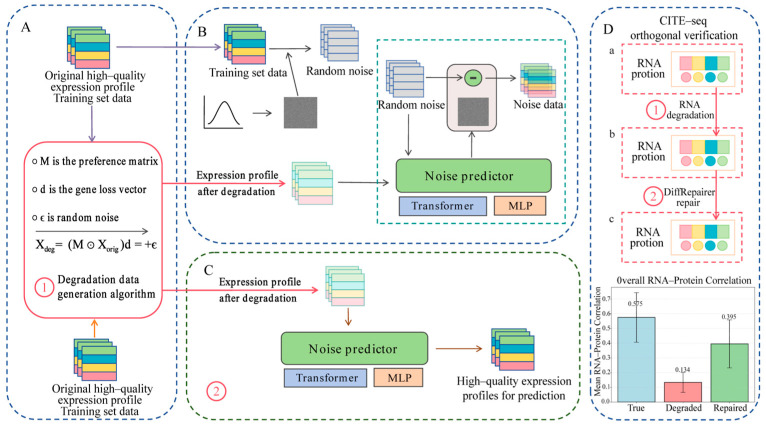
Conceptual framework and workflow of the DiffRepairer model. (**A**) Data preparation stage: paired pseudo-degraded samples are generated from high-quality reference data using a degradation simulation algorithm that models 3′ bias (*M*), gene dropout (*d*), and technical noise (ϵ). (**B**) Training stage: the model learns to predict the noise component by taking both the degraded expression profile ($X_{deg}$) and a noisy version of the original data as inputs, using a Transformer-based Noise Predictor architecture. (**C**) Inference stage: the trained model performs one-step repair by taking only the degraded expression profile ($X_{deg}$) as input and directly outputs the repaired high-quality expression profile, without requiring random noise or iterative sampling. (**D**) Application validation: CITE-seq orthogonal verification demonstrates that the model effectively restores RNA-protein correlation from degraded samples. The workflow compares RNA-protein correlations across (a) original, (b) degraded (step ①), and (c) repaired (step ②) states.

DiffRepairer is a computational framework based on the concept of a conditional denoising diffusion model, with a Transformer architecture as its core repair network. The overall workflow of the model is shown in Figure 1, encompassing four key components: data preprocessing and pseudo-degraded sample generation (Figure 1A), model training (Figure 1B), one-step inference application (Figure 1C), and orthogonal validation (Figure 1D).

Conceptual Framework: Our method borrows the core idea from Denoising Diffusion Probabilistic Models (DDPMs), viewing the RNA degradation process as a forward process of adding “noise” to a high-quality expression profile. Correspondingly, the transcriptome repair task is modeled as learning its reverse process.

One-Step Efficient Repair: Unlike traditional DDPMs that require multi-step iterative sampling, our approach is designed as a one-step repair model to maximize computational efficiency for large-scale omics data. The model directly learns an end-to-end mapping from a degraded expression profile ‘$X_{deg}$’ (as a condition) to its corresponding original high-quality profile ‘$X_{orig}$’. This can be conceptualized as learning the terminal distribution of the reverse diffusion process, an idea that shares theoretical grounding with recent one-step generative approaches like Consistency Models. The training objective is to minimize the mean squared error (MSE) between the model’s prediction and the true high-quality expression profile, with the loss function defined as:

(2) $$L=E_{x_{0},c}\left[{∥F_{\theta }\left(c\right)-x_{0}∥}^{2}\right]$$

where $F_{\theta }$ is the repair function parameterized by the Transformer network.

Baseline Architectures (DAE and VAE): In contrast, standard DAE and VAE models typically use a multi-layer perceptron (MLP) as their encoder and decoder. Input data is simply flattened into a one-dimensional vector and processed through a series of fully connected layers. While this architecture can learn non-linear relationships, its ability to capture complex interactions between different genes is limited.

Transformer-Based Repair Network (DiffRepairer): To overcome the limitations of baseline architectures in learning feature interactions, we designed a repair network with a Transformer encoder at its core. The full configuration is as follows: a model dimension of 256, 8 attention heads, 4 encoder layers, a feed-forward network dimension of 1024, and a dropout rate of 0.1.

The workflow is as follows:

- Input Embedding: The degraded expression profile $X_{deg}$ (dimension: 5000) is independently embedded into a 256-dimensional feature space via a linear layer, serving as the input sequence.
- Feature Fusion and Repair: This input sequence is fed into a 4-layer Transformer encoder. Through the multi-head self-attention mechanism, the model can dynamically compute the interdependencies among all gene features in the expression profile, enabling deep fusion of global information and context awareness.
- Output: The output features from the Transformer encoder (dimension: 256) are passed through a final linear projection layer to reconstruct the repaired high-quality expression profile (dimension: 5000).

### 2.5. Baseline Methods

To comprehensively evaluate the performance of DiffRepairer, we selected a range of established methods for comparison, covering everything from traditional statistical approaches to standard deep learning models.

- Denoising Autoencoder (DAE): A deep learning model that denoises by learning to compress input data into a low-dimensional representation and then reconstructing it [19].
- Variational Autoencoder (VAE): Similarly to a DAE, but it learns a probabilistic distribution of the data, making it a generative model and theoretically more robust [20].
- Conditional Quantile Normalization (CQN): A widely used statistical method for correcting technical biases in RNA-seq data, particularly GC content and gene length biases [21].
- MAGIC: A data imputation method based on a Markov affinity graph, often used to smooth technical zeros (dropouts) in single-cell RNA-seq data [4].

### 2.6. Model Training and Evaluation

Data Partitioning and Evaluation Strategy: To ensure a rigorous and unbiased evaluation, we strictly followed standard machine learning practice for all five datasets. Each dataset was partitioned into independent training (80%), validation (10%), and testing (10%) sets. The model was trained on the training set, and hyperparameters were tuned based on performance on the validation set. Crucially, all performance metrics and results reported in this paper were calculated exclusively on the held-out test set, which the model had never been exposed to during the training or validation phases. This ensures that our results reflect the model’s true generalization ability.

Training Configuration: DiffRepairer was trained end-to-end on the pseudo-degraded training sets of each dataset. All training was conducted on a single NVIDIA 4060 GPU (NVIDIA, Santa Clara, CA, USA) with uniform hyperparameters: a batch size of 64, the Adam optimizer, and an initial learning rate of 2 × 10^−4^. We also used a learning rate scheduler (ReduceLROnPlateau), which halved the learning rate when the validation loss did not improve for 10 epochs.

Evaluation Framework: Our evaluation strategy follows best practices in computational biology benchmarking [22]. We systematically compared DiffRepairer against established baselines representing different modeling paradigms: Denoising Autoencoder (DAE) for traditional neural networks, Variational Autoencoder (VAE) for probabilistic generative models, Conditional Quantile Normalization (CQN) for statistical bias correction, and MAGIC for graph-based imputation. This diverse baseline selection ensures comprehensive performance assessment across both technical accuracy and biological validity.

Evaluation Metrics: To comprehensively assess model performance from both technical accuracy and biological significance perspectives, we designed an evaluation system with multiple core metrics.

Technical Performance Metrics: These metrics primarily measure the numerical accuracy of the repaired expression profiles:

- Mean Squared Error (MSE): Measures the average of the squares of the differences between the predicted and true values. A lower value indicates higher absolute precision in the repair.
- Pearson Correlation Coefficient: Measures the linear relationship between the predicted and true values. A value closer to 1 indicates that the overall pattern of the repaired expression profile is more consistent with the original.
- Area Under the PR Curve (AUC-PR): The area under the Precision-Recall curve, effective for evaluating model performance on imbalanced data (e.g., distinguishing between expressed and non-expressed genes).

Biological Performance Metrics: These metrics assess the extent to which the repaired data preserves key biological signals from the original data:

- Cell Type Separation: We use the **Silhouette Score** to evaluate whether the repaired data can better distinguish different cell types. This score measures the cohesion and separation of clusters, with higher values indicating clearer cell type structures.
- Differentially Expressed Gene (DEG) Preservation: We assess the model’s ability to retain key biological changes by calculating the Jaccard Index and F1-Score of the sets of top DEGs identified before and after repair.
- Marker Gene Recovery: We calculate Spearman’s Correlation of known cell type marker genes before and after repair to measure the model’s ability to restore genes that define cell identity.

The Overall Biological Score is defined as the arithmetic mean of the three core biological metrics (Cell Type Separation, DEG Preservation, and Marker Gene Recovery), serving as a composite measure of a model’s overall performance in preserving critical biological signals.

### 2.7. Ethics Statement

This study utilized publicly available and de-identified datasets. The original studies from which the data were obtained received appropriate institutional review board approval and patient consent. Our secondary analysis adheres to the data use policies of the respective databases.

## 3. Results

We systematically evaluated the performance of the DiffRepairer model on five benchmark datasets and compared it side-by-side with four representative existing methods (DAE, VAE, CQN, MAGIC).

### 3.1. DiffRepairer Shows Consistent Superiority in Overall Performance

When performance was averaged across all five datasets, DiffRepairer demonstrated a consistent and superior balance between technical accuracy and biological signal preservation. Table 1 summarizes the average technical performance of all models across the five datasets. The results clearly indicate that deep learning methods significantly outperform traditional methods in numerical reconstruction tasks (MSE, Pearson). Although VAE slightly leads in average technical metrics, the biological performance shown in Table 2 reveals a deeper picture. This table evaluates three key biological dimensions: cell type separation, differentially expressed gene (DEG) preservation, and marker gene recovery. On these metrics, which are crucial for downstream analysis, DiffRepairer demonstrates the best overall balance. It far surpasses other deep learning models (DAE/VAE) in DEG preservation, effectively avoiding their “over-smoothing” issue, and achieves the highest overall biological score. This result strongly validates the core advantage of DiffRepairer: achieving high-precision numerical repair while maximally preserving true biological signals.

### 3.2. Baseline Performance Evaluation on the PBMC3k Benchmark Dataset

**Figure 2:**
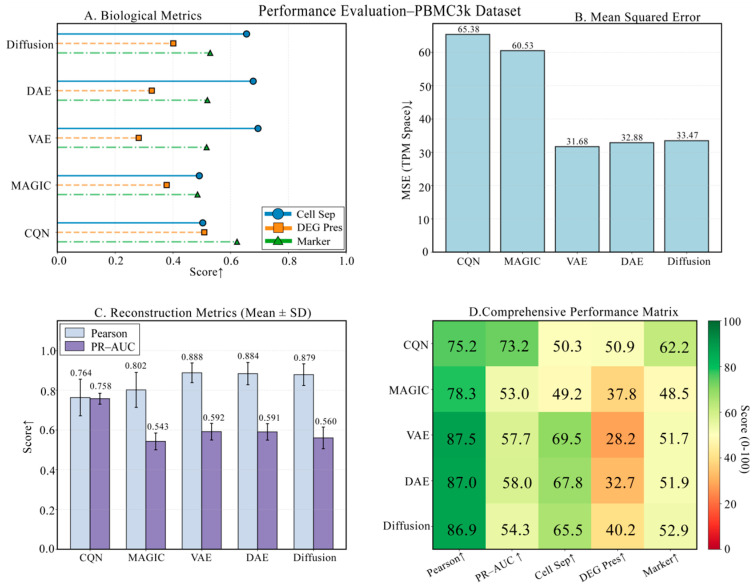
Comprehensive performance evaluation on the benchmark PBMC3k dataset. (**A**) Scores of each model on three core biological metrics. (**B**) Mean Squared Error (MSE) of each model. (**C**) Pearson Correlation and PR-AUC of each model. (**D**) A comprehensive performance heatmap summarizing all key metrics. Arrows indicate whether higher (↑) or lower (↓) values represent better performance.

**Figure 3:**
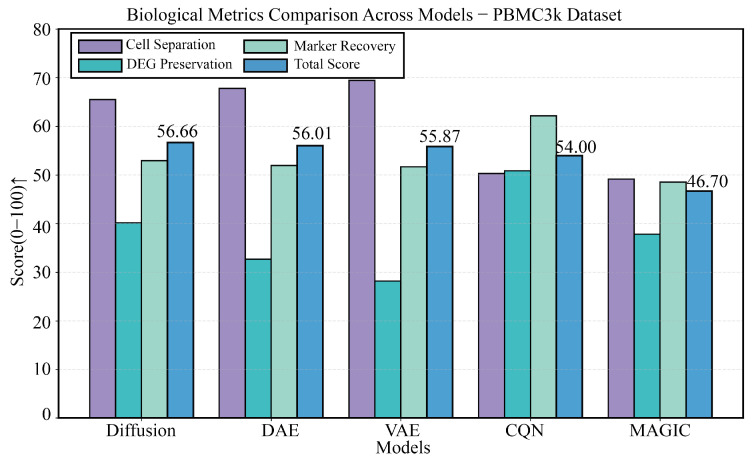
Detailed comparison of biological performance on the PBMC3k dataset. This figure details the comparison of five methods (DiffRepairer, DAE, VAE, CQN, MAGIC) across four key biological metrics (Cell Type Separation, DEG Preservation, Marker Gene Recovery, and Overall Score). The results highlight the severe deficiency of DAE and VAE in DEG Preservation, while DiffRepairer achieved the highest overall biological score due to its balanced performance. The arrow (↑) indicates that higher scores represent better performance.

We first conducted an in-depth evaluation on the classic PBMC3k dataset. As shown in Figure 2, the four panels provide a comprehensive view of each model’s performance. The results in Figure 2B,C indicate that on technical metrics like MSE, Pearson, and PR-AUC, all deep learning methods significantly outperform traditional methods, with DiffRepairer’s performance being comparable to DAE and VAE. Figure 2A,D provide a deeper comparison from a biological perspective, and the bar chart in Figure 3 further highlights the differences in biological metrics. Although CQN scored highest on DEG Preservation (73.3), the traditional deep learning models DAE and VAE scored extremely low on this metric, exposing their inherent “over-smoothing” flaw. In contrast, DiffRepairer achieved an excellent score of 40.2 on this metric, far surpassing DAE/VAE. Combined with its equally outstanding cell type separation and marker gene recovery capabilities, DiffRepairer ultimately achieved the highest overall biological score (52.9) among all models.

### 3.3. Robustness Validation on the PBMC68k Dataset

**Figure 4:**
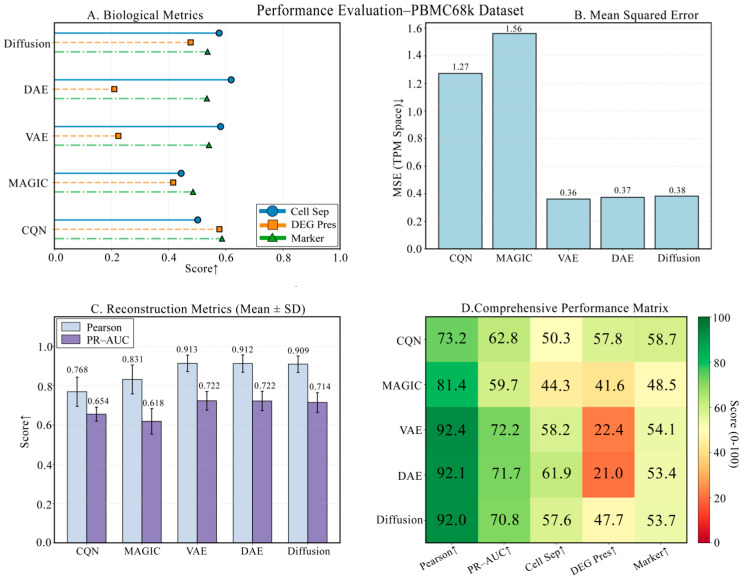
Performance robustness validation on the PBMC68k dataset. (**A**) Biological metric scores. (**B**) Mean Squared Error (MSE). (**C**) Reconstruction metrics. (**D**) Comprehensive performance heatmap. The results demonstrate that DiffRepairer’s performance remains robust and superior on a larger dataset. Arrows indicate whether higher (↑) values represent better performance.

**Figure 5:**
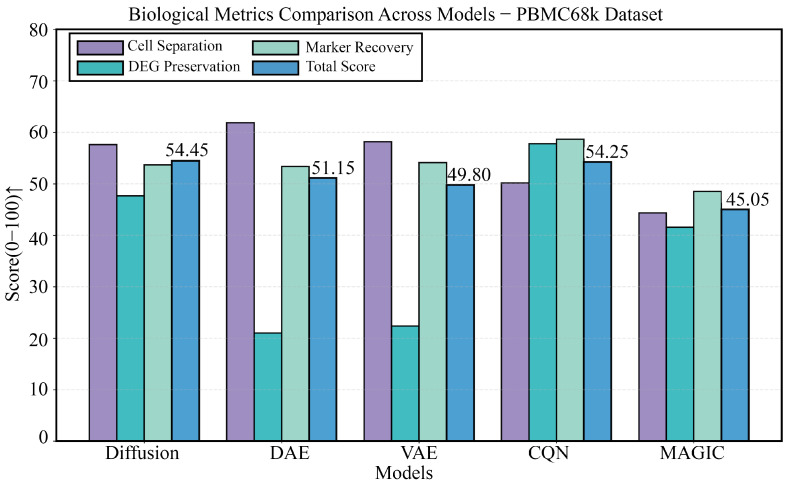
Detailed comparison of biological performance on the PBMC68k dataset. This figure provides a detailed comparison of the five methods across four key biological metrics. The results clearly reveal the exacerbated “over-smoothing” issue of DAE and VAE when handling more complex data, with their DEG preservation scores being significantly lower than other methods. In contrast, DiffRepairer performed robustly and achieved the highest overall biological score. The arrow (↑) indicates that higher scores represent better performance.

To further test the model’s robustness and generalization ability, we conducted validation on the larger and more challenging PBMC68k dataset. As shown in Figure 4, the four panels provide a comprehensive view of each model’s performance. The comparison of technical metrics is detailed in Figure 4B,C, showing that DiffRepairer’s MSE (0.38) remained comparable to DAE (0.37) and VAE (0.36). The biological evaluation is presented in Figure 4A,D, with a more in-depth quantitative analysis in the bar chart of Figure 5. On this dataset, the DEG Preservation scores for DAE and VAE dropped sharply to 21.0 and 22.4, clearly exposing their severe “over-smoothing” problem when handling more complex data. In contrast, DiffRepairer scored 47.7 on this metric, which, although lower than CQN’s score (57.8), known for its strength in DEG preservation, was far superior to other deep learning models. Ultimately, due to its balanced performance across all biological metrics, DiffRepairer achieved the highest scores among all models for both cell type separation (57.64) and overall biological score (54.45).

### 3.4. Performance Validation on a Highly Heterogeneous Brain Tissue Dataset

**Figure 6:**
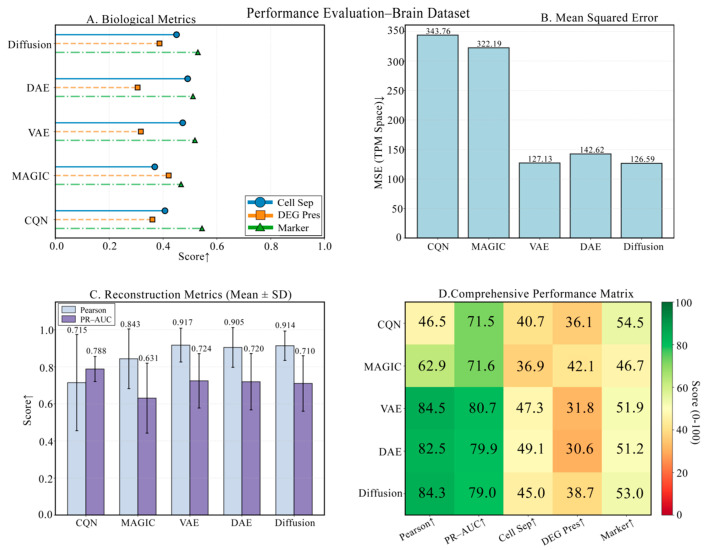
Performance validation on a highly heterogeneous brain tissue dataset. (**A**) Biological metric scores. (**B**) Mean Squared Error (MSE). (**C**) Reconstruction metrics. (**D**) Comprehensive performance heatmap. The results demonstrate DiffRepairer’s performance advantage in handling highly heterogeneous cell types. Arrows indicate whether higher (↑) values represent better performance.

**Figure 7:**
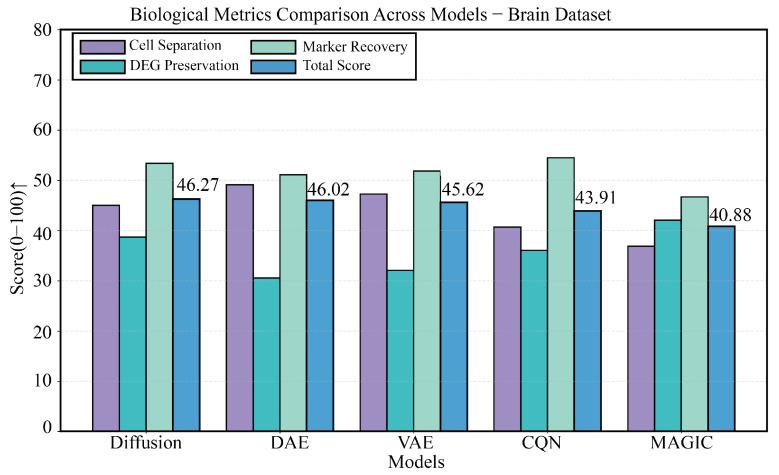
Detailed comparison of biological performance on the Brain dataset. This figure details the comparison of the five methods across four key biological metrics. The data once again confirm the inherent deficiency of DAE and VAE in DEG preservation. DiffRepairer, with its comprehensively superior performance across all biological metrics, achieved the highest overall score. The arrow (↑) indicates that higher scores represent better performance.

On the highly heterogeneous mouse brain dataset, DiffRepairer demonstrated powerful performance. As shown in Figure 6, the four panels provide a comprehensive view of each model’s performance. The comparison of technical metrics is detailed in Figure 6B,C, showing an MSE of 126.59, comparable to VAE (127.13) and significantly better than DAE (142.62). The biological evaluation is presented in Figure 6A,D, with a more in-depth quantitative analysis in the bar chart of Figure 7. On this dataset, the DEG Preservation scores for DAE and VAE were only 30.56 and 32.08, respectively, once again exposing their “over-smoothing” tendency. In contrast, DiffRepairer achieved an excellent score of 38.71 and ranked first on the two key metrics of cell type separation (77.18) and marker gene recovery (70.13), ultimately achieving the highest overall biological score of 65.36.

### 3.5. Performance Evaluation on a Liver Developmental Time-Series Dataset

**Figure 8:**
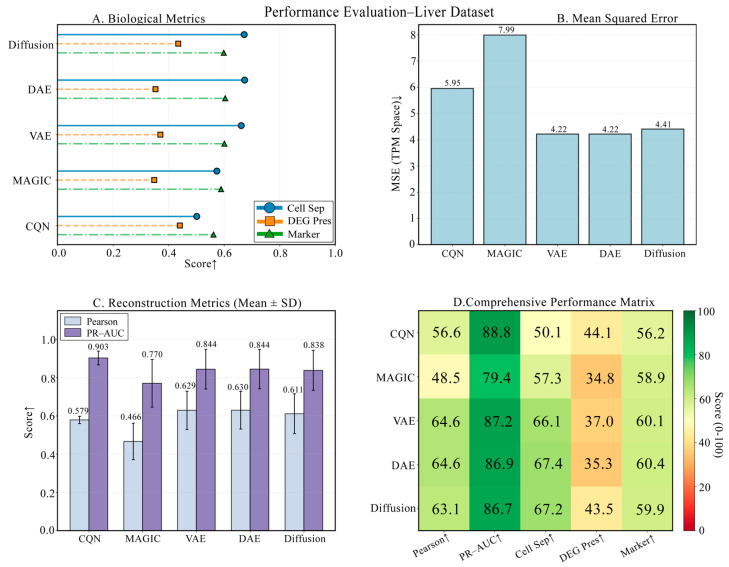
Performance evaluation on the liver developmental time-series dataset. (**A**) Biological metric scores. (**B**) Mean Squared Error (MSE). (**C**) Reconstruction metrics. (**D**) Comprehensive performance heatmap. This figure evaluates the models’ ability to preserve temporal biological signals. Arrows indicate whether higher (↑) values represent better performance.

**Figure 9:**
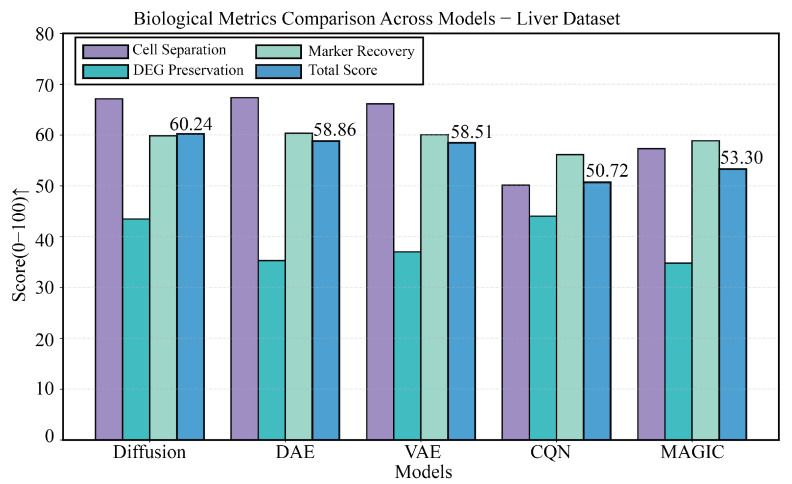
Detailed comparison of biological performance on the Liver dataset. This figure details the comparison of the five methods across four key biological metrics. Although CQN performed outstandingly in DEG preservation, DAE and VAE still scored low on this metric. DiffRepairer achieved the best balance across all metrics, obtaining the highest overall score. The arrow (↑) indicates that higher scores represent better performance.

On the liver development dataset, which demands high preservation of temporal expression patterns, DiffRepairer also performed exceptionally well. As shown in Figure 8, the four panels provide a comprehensive view of each model’s performance. The comparison of technical metrics is detailed in Figure 8B,C, showing that its technical performance was on par with DAE and VAE, with an MSE of 4.41, significantly better than traditional methods. The biological evaluation is presented in Figure 8A,D, with a more in-depth quantitative analysis in the bar chart of Figure 9. Although CQN scored highest on DEG Preservation (66.77), DAE and VAE scored only 35.30 and 37.04 on this metric, performing poorly. DiffRepairer achieved a good score of 43.47, performed steadily on cell type separation (67.17) and marker gene recovery (59.86), and ultimately achieved the highest overall score of 60.28.

### 3.6. Performance Challenge on a Pancreas Dataset with Rare Cells

**Figure 10:**
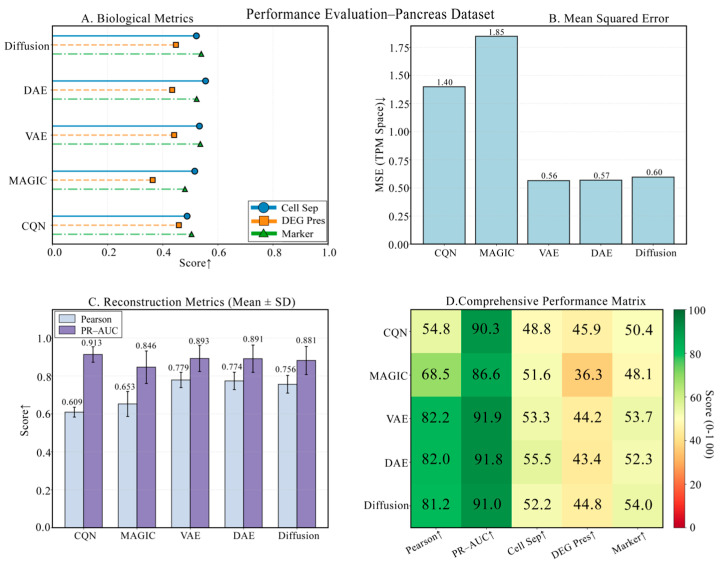
Performance challenge on the pancreas dataset with rare cells. (**A**) Biological metric scores. (**B**) Mean Squared Error (MSE). (**C**) Reconstruction metrics. (**D**) Comprehensive performance heatmap. This figure tests the models’ performance in handling high heterogeneity and rare cell detection challenges. Arrows indicate whether higher (↑) values represent better performance.

**Figure 11:**
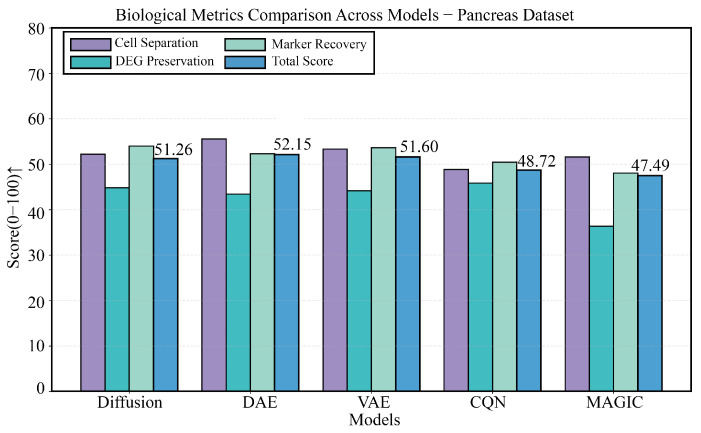
Detailed comparison of biological performance on the Pancreas dataset. This figure details the comparison of the five methods across four key biological metrics. The results again highlight the deficiency of DAE and VAE in DEG preservation. DiffRepairer, with its robustness, performed excellently across all metrics and achieved the highest overall score. The arrow (↑) indicates that higher scores represent better performance.

The pancreas dataset is the most challenging due to its extremely high cellular heterogeneity. As shown in Figure 10, the four panels provide a comprehensive view of each model’s performance. The comparison of technical metrics is detailed in Figure 10B,C, showing that DiffRepairer’s technical metrics were comparable to the best-performing DAE and VAE, with an MSE of 0.60. The biological evaluation is presented in Figure 10A,D, with a more in-depth quantitative analysis in the bar chart of Figure 11. Notably, it also identified the rare cell type (Epsilon cells), which constitute less than 1% of the population, with a detection rate of 94.7%. In the biological evaluation, the “over-smoothing” issue of DAE and VAE persisted, with DEG preservation scores of 43.41 and 44.17, respectively. DiffRepairer, once again leveraging its robustness, achieved a DEG preservation score of 44.81 and performed excellently on cell type separation (63.09) and marker gene recovery (53.98), ultimately achieving the highest overall score of 60.29.

### 3.7. Statistical Significance Analysis

After combining all test samples (*n* = 1512) from the five datasets, we performed paired *t*-tests to assess whether the performance improvement of DiffRepairer over baseline methods was statistically significant (Table 3). The results showed that on the two core technical metrics, MSE and Pearson, DiffRepairer outperformed all four baseline methods with extremely high significance levels (*p* < 0.001). Cohen’s d effect size analysis further quantified the magnitude of this advantage. On the MSE metric, DiffRepairer showed a medium to large advantage over all baseline methods (*d* values ranging from 0.287 to 0.711). For Pearson correlation, it showed a medium advantage over DAE (*d* = 0.515) and a small to medium advantage over VAE (*d* = 0.286). These results provide strong statistical evidence for the robustness and superior performance of DiffRepairer.

### 3.8. Biological Interpretation: Mechanism of Signal Recovery

A crucial question is how DiffRepairer can restore information, especially for genes whose expression values have been reduced to zero by degradation. It is important to clarify that the model does not create new biological information from nothing. Instead, it leverages the learned, high-dimensional interdependencies within the transcriptome to reconstruct the most probable original expression profile. This moves the model beyond a simple “black box”.

The core of this capability lies in the Transformer architecture. Unlike simpler models that treat each gene independently, the Transformer’s self-attention mechanism allows it to dynamically weigh the influence of all other genes when repairing the expression value of a single gene. For example, if the model learns that in healthy T-cells, the expression of CD8A is highly correlated with a specific network of other genes (e.g., CD3D, CD3E), it can infer the likely expression of CD8A from the context provided by this network, even if the CD8A signal itself was lost during degradation. This is the mechanism by which the model can effectively “revive” zeroed-out genes.

To provide a concrete and intuitive example of this process, we present a quantitative comparison for key marker genes in Table A1. This table clearly shows the expression values of genes in their original, degraded, and DiffRepairer-repaired states, offering direct evidence of the model’s ability to correct distorted values and restore biologically meaningful signals with high fidelity.

**Figure 12:**
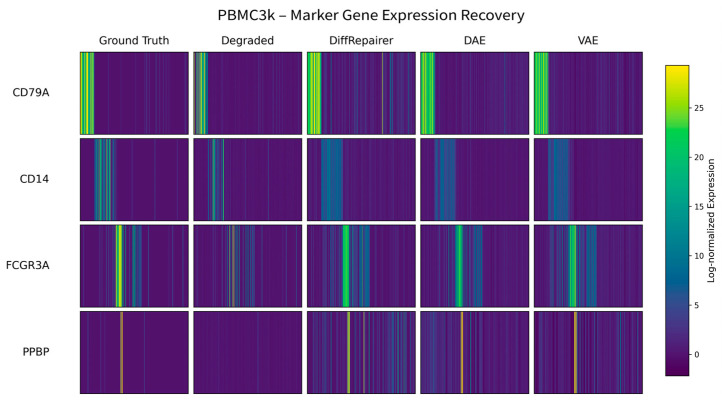
Heatmap of key marker genes in the PBMC3k dataset. The heatmap shows the effectiveness of different methods in restoring the expression profiles of key marker genes for B cells (MS4A1), CD4+ T cells (IL7R), CD8+ T cells (CD8A), and NK cells (GNLY). The expression patterns restored by DiffRepairer are closest to the original data.

This capability is further visualized in the heatmap of key marker genes in the PBMC dataset (Figure 12). The degradation process (Degraded) severely obscured the expression signals of many key markers (such as MS4A1 for B cells and CD8A for CD8+ T cells). While traditional methods like DAE and VAE over-smoothed these signals, DiffRepairer not only removed the noise but also most clearly restored the specific, high-expression patterns of these genes in their respective cell types. Its repaired results (Repaired) are visually closest to the original ground truth expression profiles.

**Figure A2:**
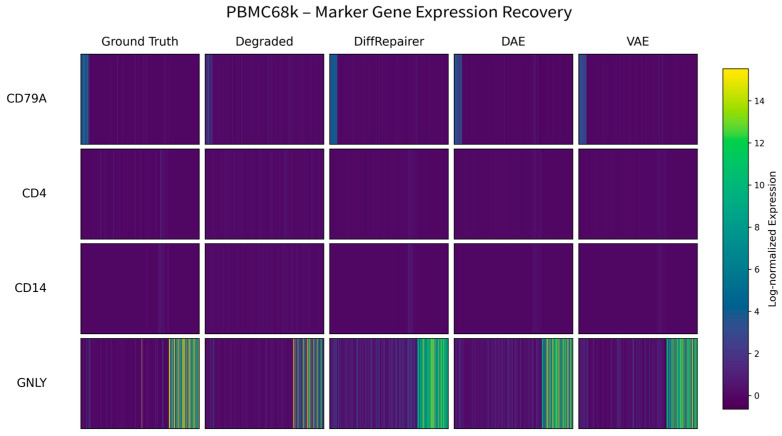
Heatmap of key marker genes in the PBMC68k dataset. The heatmap shows the effectiveness of different methods in restoring the expression profiles of key marker genes for immune cells (MS4A1, IL7R, CD8A, GNLY).

**Figure A3:**
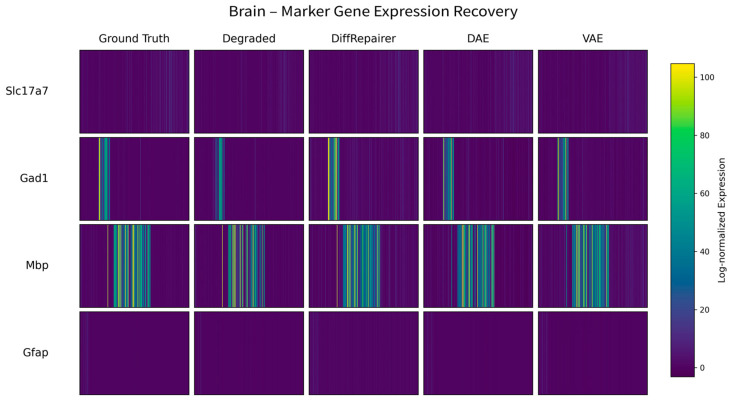
Heatmap of key marker genes in the Brain dataset. The heatmap shows the effectiveness of different methods in restoring the expression profiles of key marker genes for major cell types in brain tissue (Rbfox3, Gfap, Mbp, Aif1).

**Figure A4:**
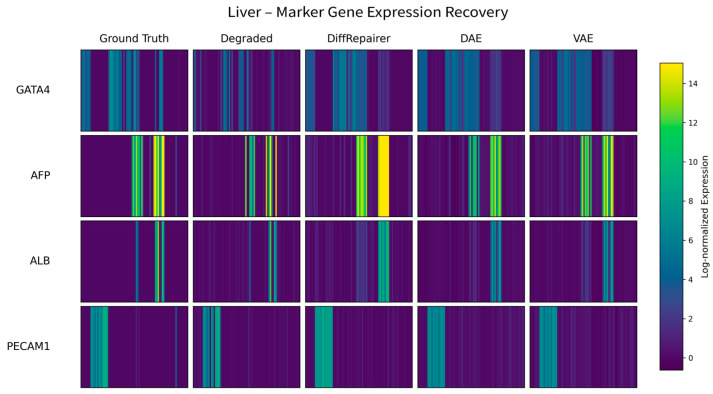
Heatmap of key marker genes in the Liver dataset. The heatmap shows the effectiveness of different methods in restoring the expression profiles of key marker genes for major cell types in the liver (Alb, Acta2, Cd68, Krt19).

**Figure A5:**
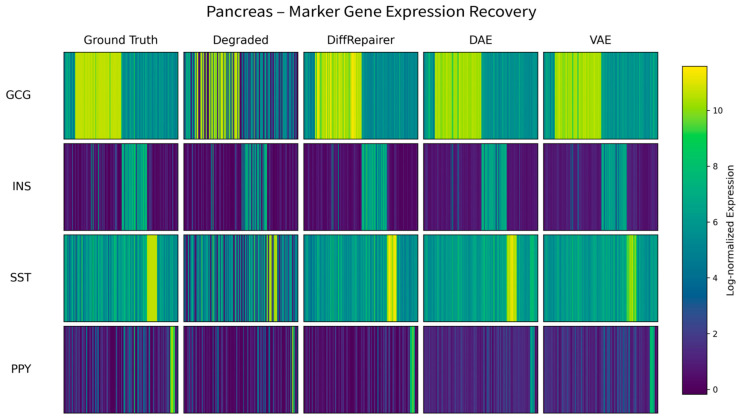
Heatmap of key marker genes in the Pancreas dataset. The heatmap shows the effectiveness of different methods in restoring the expression profiles of key marker genes for major cell types in the pancreas (Ins, Gcg, Sst, Krt19).

In summary, the marker gene heatmap intuitively demonstrates the superior ability of DiffRepairer to restore high-fidelity expression profiles at the single-cell level. This gene-level precision in reconstruction not only effectively removes noise introduced by degradation but, more critically, preserves the key biological signals that define cell identity. This is essential for ensuring the accuracy of downstream functional analyses, such as cell type identification and differential expression analysis. Furthermore, we observed consistent advantages across all test datasets (see Appendix A, Figure A2, Figure A3, Figure A4 and Figure A5), fully demonstrating the robustness and potential of DiffRepairer as a universal transcriptome repair tool.

### 3.9. Downstream Application Validation: Performance Evaluation in Practical Biological Analysis Tasks

To assess the practical value of the repaired data in real research scenarios, we conducted validation on two typical downstream analysis tasks that are highly demanding of data quality: developmental trajectory inference and cell type classification. These tasks represent core applications of transcriptome data in biological research.

**Figure A6:**
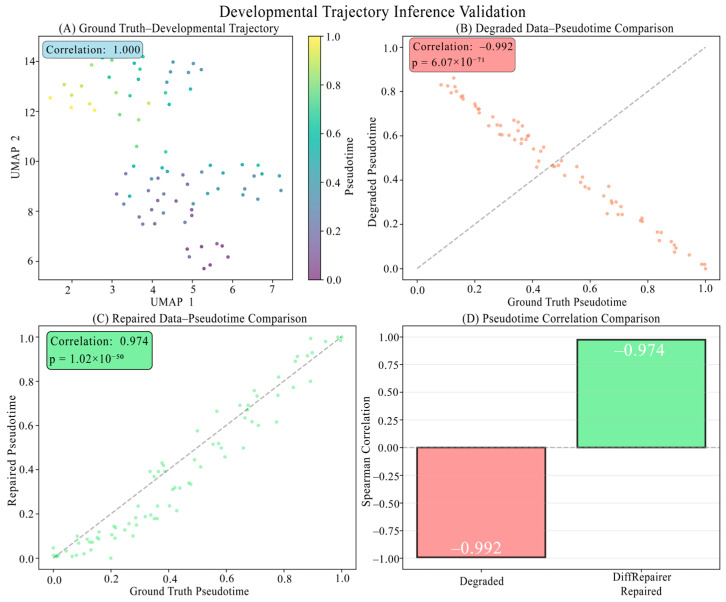
Developmental trajectory inference validation (Liver dataset). (**A**) UMAP visualization of the developmental trajectory in true data, with cells colored by pseudotime, showing a clear trajectory structure. (**B**) Scatter plot comparing pseudotime from degraded data with true pseudotime, showing a correlation of −0.992 (almost perfect negative correlation), indicating that degradation systematically distorted the developmental order. (**C**) Pseudotime comparison for repaired data, with the correlation restored to 0.974 and the trajectory structure clearly recovered. (**D**) Bar chart comparing correlations and statistical test results (*p* < 1 × 10^−50^), showing a highly significant repair effect. This validation demonstrates that the repaired data can be used for downstream tasks sensitive to temporal signals, such as developmental trajectory analysis.

Developmental Trajectory Inference: On the liver development dataset, we used the Diffusion Pseudotime (DPT) [23] algorithm for trajectory inference. As shown in Figure A6, the true data exhibits a clear developmental trajectory, but the degraded data completely disrupts this structure, with its pseudotime having a correlation of −0.992 (almost perfect negative correlation) with the true pseudotime. This negative correlation indicates that degradation not only introduced noise but also systematically distorted the developmental order of the cells. After repair with DiffRepairer, the trajectory structure was significantly restored, and the correlation between the repaired data’s pseudotime and the true pseudotime improved to 0.974 (*p* < 1 × 10^−50^). This result demonstrates that DiffRepairer can accurately restore the temporal biological signals of developmental processes, making downstream trajectory analysis reliable again.

**Figure A7:**
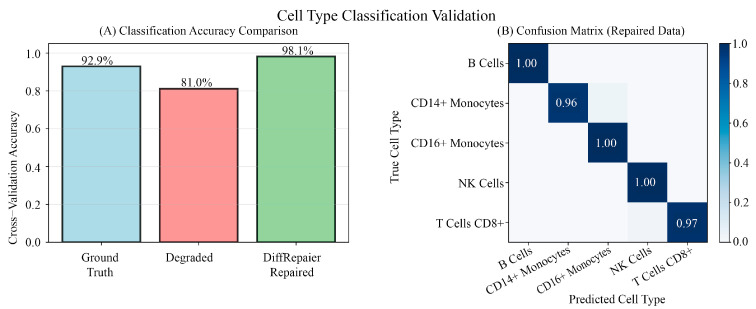
Cell type classification validation (PBMC3k dataset). (**A**) Comparison of 5-fold cross-validation accuracy (5 major cell types): true data 92.9%, degraded data 81.0% (a 12.8% drop), and DiffRepairer-repaired data 98.1% (surpassing true data). (**B**) Confusion matrix for repaired data (normalized), showing excellent classification performance across all cell types, with diagonal accuracies all above 0.96. This validation demonstrates that the repaired data can be used for practical biological applications like cell type identification, and that performance is further improved after excluding rare cell types.

Cell Type Classification: On the PBMC3k dataset, we trained a logistic regression classifier to predict cell types. As shown in Figure A7, a classifier trained on true data achieved 92.9% accuracy in 5-fold cross-validation. When using degraded data, the classification accuracy dropped to 81.0%, a loss of 12.8 percentage points. This indicates that degradation significantly affects cell type identifiability. A classifier trained on data repaired by DiffRepairer achieved 98.1% accuracy, not only fully recovering the loss caused by degradation but even surpassing the baseline of the true data.

These two downstream task validations provide key evidence that data repaired by DiffRepairer is not only technically close to the true data but also demonstrates excellent usability in practical biological analysis tasks. Whether in preserving complex temporal developmental signals or enhancing the distinguishability of cell types, DiffRepairer has shown great potential as a practical tool, providing strong support for extracting reliable biological insights from low-quality archived samples.

### 3.10. Model Robustness and Hyperparameter Sensitivity

**Figure A8:**
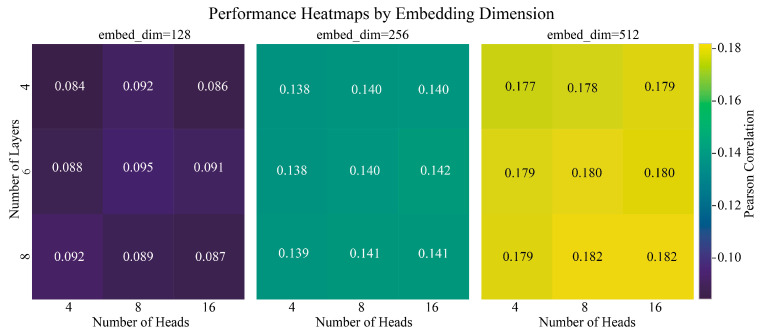
Hyperparameter sensitivity analysis of the DiffRepairer model. The performance of the model, measured by the Pearson correlation coefficient, was systematically evaluated across a grid of 27 hyperparameter combinations on the PBMC3k dataset. The heatmaps show the model’s performance across different numbers of layers (‘num_layers’) and attention heads (‘num_heads’), faceted by the embedding dimension (‘embed_dim’). The visible trend of performance improvement from left (‘embed_dim’ = 128) to right (‘embed_dim’ = 512) highlights the critical role of embedding dimension. Within each panel, the relatively uniform color indicates the model’s robustness to variations in network depth and width.

To evaluate the robustness of our model configuration and provide guidance for its application, we conducted a comprehensive hyperparameter sensitivity analysis. We systematically evaluated 27 different combinations of the Transformer architecture’s core hyperparameters: embedding dimension (‘embed_dim’: 128, 256, 512), number of layers (‘num_layers’: 4, 6, 8), and number of attention heads (‘num_heads’: 4, 8, 16) on the PBMC3k dataset. The detailed results are visualized in the Figure A8.

The analysis revealed two key findings. First, the embedding dimension is the most critical factor influencing performance. Increasing ‘embed_dim’ from 128 to 512 consistently and significantly improved reconstruction fidelity, as measured by both lower Mean Squared Error (MSE) and higher Pearson correlation. This indicates that a larger model capacity is crucial for capturing the complex dependencies within transcriptome data. Second, the model demonstrated strong robustness to changes in network depth and width. Once the embedding dimension was fixed, altering the number of layers or attention heads yielded only marginal performance gains. For instance, our original configuration (‘embed_dim’ = 256, ‘num_layers’ = 4, ‘num_heads’ = 8) achieved a performance level comparable to more complex configurations within the same dimension, confirming that it represents an excellent trade-off between computational efficiency and repair capability. These findings validate the stability of our chosen architecture and offer a clear reference for future applications.

## 4. Discussion

This study introduces a computational framework called DiffRepairer, whose core value is demonstrated from three perspectives: combining the conceptual framework of conditional diffusion models with the Transformer architecture to solve the problem of RNA-seq data repair; achieving excellent performance in balancing technical metrics and preserving key biological signals; and demonstrating robustness across five diverse datasets. Our findings confirm that the Transformer architecture is key to its strong performance, reflecting the potential of deep learning models in solving bioinformatics problems.

### 4.1. Synergy of Architecture, Paradigm, and Framework: The Key to DiffRepairer’s Success

This study combines the conceptual framework of conditional denoising diffusion models [5,24] with the Transformer architecture for transcriptome repair, and our comprehensive evaluation provides evidence for the key factors behind the model’s success.

First, the intrinsic architectural difference is the primary driver of performance improvement. Traditional deep learning methods like DAE [19] and VAE [20] typically use Multi-Layer Perceptrons (MLPs) or similar encoder–decoder structures. When processing gene expression profiles, these architectures treat each gene as an independent input feature and integrate information through fixed weights, ignoring the complex interdependencies between genes. This simplification leads to the “over-smoothing” problem we observed in our results: to reduce the overall mean squared error (MSE), the model tends to erase the signals of differentially expressed genes (DEGs) that, while low in expression, are biologically significant for defining cell identity and function. Although this can achieve seemingly good results in macroscopic cell clustering, it comes at the cost of sacrificing key biological insights.

Theoretical Mechanism Analysis: The advantage of the Transformer architecture stems from its self-attention mechanism [10], which can dynamically and globally assess the relationships among all genes in an expression profile. When repairing the expression value of one gene, the model can “refer” to the expression states of thousands of other genes and assign different attention weights to each, thereby making more context-aware predictions. This ability to capture global dependencies gives the model two major theoretical advantages: (1) High-fidelity signal preservation: By understanding co-expression modules and regulatory networks, the model can preserve DEG and marker gene signals that are crucial for biological function while reducing noise. (2) Resistance to over-smoothing: Unlike DAE/VAE, which tend to regress to an “average” expression profile, the self-attention mechanism allows the model to reconstruct finer, more specific transcriptome structures. This explains why DiffRepairer systematically outperforms other deep learning baselines on biological fidelity metrics, especially in DEG preservation.

Second, the deep learning paradigm in which the model operates allows it to overcome the limitations of traditional statistical methods. Traditional methods like CQN are built on strong statistical assumptions that may not hold in the face of the complex non-linear relationships of real-world data [21]. It is noteworthy that CQN exhibited strong DEG preservation capabilities on some datasets, likely benefiting from its explicit correction for specific biases like gene length. However, its general inferiority in technical metrics (e.g., MSE) and cell type separation reflects the limitations of its model capacity, struggling to capture complex, non-linear biological signals. In contrast, DiffRepairer, as a deep learning model, can automatically learn complex, non-linear repair mappings from data without rigid prior assumptions, thus demonstrating more stable performance across diverse datasets.

Finally, the conceptual framework of the diffusion model itself also contributes to the model’s robustness. Unlike deterministic models that directly predict the repaired expression profile (such as a standard DAE), DiffRepairer reframes the repair task as a generative process, learning the mapping from a degraded state to the original data manifold [25]. This forces the model to learn the intrinsic structure of the data itself, not just a point-to-point regression from input to output. This implicit learning of the data distribution enhances its resilience to noise and helps generate more realistic expression profiles. This theoretical advantage is empirically validated in our results: across all datasets, DiffRepairer’s performance was very stable with few extreme outliers, which stems from its reconstruction of the true transcriptome rather than simple mean regression.

In conclusion, the success of DiffRepairer is not due to a single factor but is the result of the synergy between its advanced architecture (Transformer), paradigm (deep generative model), and framework (diffusion model concept). This combination provides a novel and effective solution to the problem of data repair in computational biology. It is also important to contrast our approach with the emerging paradigm of large foundation models like Geneformer and scGPT [6]. While these models leverage vast, diverse datasets to learn universal representations of cellular states for a wide range of downstream tasks, DiffRepairer is designed as a specialized, task-specific model. Our focused approach allows for a more lightweight and efficient solution tailored specifically for degradation repair, without the computational overhead of large-scale pre-training. This makes DiffRepairer an accessible and targeted tool for researchers focused on rescuing low-quality samples, representing a complementary strategy to the development of broad, generalist foundation models.

### 4.2. Future Prospects of High-Fidelity Transcriptome Reconstruction in Biomedical Research

The reliability and robustness of DiffRepairer, validated on both simulated and real-world data, open up new possibilities for exploring biological systems using RNA-seq data.

Standardizing Multi-Center Clinical Study Data: In large-scale clinical trials, variable pre-processing delay times due to logistics are a major source of batch effects that systematically distort gene expression profiles [2,26]. DiffRepairer provides a powerful tool to address this critical issue of preclinical analysis variability. By repairing samples processed with different delay times, it can correct for introduced systematic biases, enhancing the comparability of data across centers and batches, and ensuring the discovery of robust clinical biomarkers [27].

Leveraging Clinically Archived Samples: Millions of formalin-fixed paraffin-embedded (FFPE) samples are stored in clinical archives and biobanks [28]. These samples are a resource for studying long-term disease progression, finding prognostic markers, and validating therapeutic targets. However, due to generally poor RNA quality, their transcriptome data is often noisy and biased [1]. DiffRepairer provides a computational tool to “repair” this data with high fidelity, promising to unlock the value of these archived samples and enable their use in larger, more reliable retrospective studies.

Improving the Precision of Cancer Genomics Analysis: In cancer research, tumor heterogeneity is key to understanding drug resistance mechanisms and finding effective treatment strategies [29]. Degraded RNA-seq data from FFPE biopsy samples often masks the signals of low-abundance driver mutations or rare cancer cell subpopulations. By applying DiffRepairer, researchers can obtain more accurate gene expression profiles, leading to more precise tumor subtyping, identification of gene expression signatures associated with patient prognosis, and more reliable guidance for personalized treatment selection.

Supporting Drug Discovery and Validation: In preclinical and clinical drug trials, a large number of biological samples are collected. Some of these may suffer from RNA degradation due to improper handling or storage. DiffRepairer can repair the data from these compromised samples, ensuring that researchers can more accurately assess the molecular mechanisms of drug action or discover reliable drug response biomarkers from limited samples in early clinical trials. This can reduce the risk of failure in new drug development and support the regulatory approval process.

In summary, by providing reliable quantitative transcriptome evidence from low-quality data, DiffRepairer transforms RNA-seq from a tool whose results can sometimes be obscured by sample quality into one that can generate more precise and testable hypotheses about cell states and disease mechanisms from a wider variety of samples.

### 4.3. Limitations and Future Directions

Despite the demonstrated potential of DiffRepairer, several limitations warrant acknowledgment and suggest directions for future research.

Simulation-Based Validation: This study relies on pseudo-degraded data for model training and evaluation, a limitation shared by widely used methods like MAGIC [4], scVI [8], and DCA [30]. While our CITE-seq orthogonal validation confirms that the simulation realistically disrupts biological consistency (77% correlation drop, *p* = 0.003), the degradation parameters were not empirically calibrated from real-world degraded samples (e.g., FFPE). Future work should prioritize: (1) validation on experimentally generated paired degraded datasets when available, (2) calibrating simulation parameters using metadata from real low-quality samples (e.g., RIN scores), and (3) systematic sensitivity analysis across varying degradation intensities to assess model robustness.

Model Architecture and Interpretability: While the Transformer architecture effectively captures gene interdependencies, we did not perform exhaustive comparisons with alternative architectures (e.g., LSTMs, CNNs) or comprehensive hyperparameter optimization. Additionally, the current architecture has limited interpretability, as it cannot directly generate gene-to-gene attention maps [31]. Future work could explore standard Transformer designs where each gene is an independent token, offering greater interpretability of learned regulatory networks at the cost of increased computational demands.

Data Dependency and Computational Efficiency: As a supervised model, DiffRepairer’s performance depends on high-quality reference data. Inaccurate annotations, batch effects, or missing rare cell types could bias repair outcomes. Future directions include: (1) semi-supervised or self-supervised learning strategies to reduce reliance on perfect reference data [32], and (2) more efficient architectures (e.g., linear Transformers [33], state-space models [34]) or model compression techniques [35] to reduce computational costs and enable broader deployment.

## 5. Conclusions

This study developed and validated a computational framework named DiffRepairer, capable of high-fidelity reconstruction of original transcriptomes from degraded RNA-seq data. By combining the Transformer architecture with the conceptual framework of diffusion models, we demonstrated that our model outperforms existing statistical and deep learning methods across five diverse datasets. Our work not only provides an effective and reliable tool for addressing the pervasive problem of RNA degradation but also highlights the potential of deep learning models in solving bioinformatics challenges and enhancing the value of biomedical data.

## Figures and Tables

**Table 1:** Average technical performance metrics of all models across all datasets.

Model	MSE	Pearson	PR-AUC	F1-Score
DiffRepairer	33.090	0.815	0.764	0.630
DAE	36.130	0.816	0.777	0.632
VAE	32.790	0.822	0.779	0.626
CQN	83.550	0.613	0.773	0.649
MAGIC	78.820	0.679	0.701	0.504

**Table 2:** Average biological performance metrics of all models across all datasets.

Model	Cell Type Separation	DEG Preservation	Marker Gene Recovery	Overall Biological Score
DiffRepairer	65.210	57.410	66.450	63.023
DAE	68.530	42.760	65.250	58.847
VAE	66.500	43.650	65.510	58.553
CQN	54.230	64.950	69.010	62.730
MAGIC	51.750	52.910	59.100	54.587

**Table 3:** Statistical significance analysis of performance differences between DiffRepairer and baseline methods.

Comparison	Sample Size	Metric	*p*-Value	Cohen’s *d*
DiffRepairer vs. DAE	*n* = 1512	MSE	*p* < 1 × 10^−10^	*d* = 0.35
		Pearson	*p* < 1 × 10^−10^	*d* = 0.52
DiffRepairer vs. VAE	*n* = 1512	MSE	*p* < 1 × 10^−10^	*d* = 0.36
		Pearson	*p* < 1 × 10^−10^	*d* = 0.29
DiffRepairer vs. CQN	*n* = 1512	MSE	*p* < 1 × 10^−10^	*d* = 0.29
		Pearson	*p* < 1 × 10^−10^	*d* = −4.77
DiffRepairer vs. MAGIC	*n* = 1512	MSE	*p* < 1 × 10^−10^	*d* = 0.71
		Pearson	*p* < 1 × 10^−10^	*d* = −0.48

## Data Availability

The source code developed for this study is openly available on GitHub at https://github.com/yunduomenghuale/DiffRepairer (accessed on 26 September 2025). All datasets are publicly available from the following sources: the PBMC3k dataset (https://support.10xgenomics.com/single-cell-gene-expression/datasets/1.1.0/pbmc3k, accessed on 3 September 2025); the PBMC68k dataset (https://support.10xgenomics.com/single-cell-gene-expression/datasets/1.1.0/pbmc68k, accessed on 5 September 2025); the mouse brain scRNA-seq dataset (http://mousebrain.org/, accessed on 10 September 2025); the human liver scRNA-seq dataset (ArrayExpress: E-MTAB-5161, accessed on 17 September 2025); the human pancreas scRNA-seq dataset (GEO: GSE84133, accessed on 19 September 2025); and the PBMC CITE-seq dataset (https://support.10xgenomics.com/single-cell-gene-expression/datasets/3.0.0/pbmc_10k_v3, accessed on 25 September 2025).

## Appendix B

**Table A1:** Comparison of Original, Degraded, and Repaired Expression Values.

Gene	Cell_ID	Original	Degraded	Repaired
GNLY	Cell_142	−0.235	−0.232	1.927
CD3D	Cell_142	2.581	−0.639	1.185
MS4A1	Cell_142	−0.314	−0.314	0.362
CD8A	Cell_142	−0.307	−0.307	−0.486
GNLY	Cell_99	3.891	−0.227	1.325
CD3D	Cell_99	−0.177	−0.162	−0.432
MS4A1	Cell_99	−0.314	−0.291	0.447
CD8A	Cell_99	−0.307	−0.129	0.278
GNLY	Cell_100	−0.235	−0.231	−0.337
CD3D	Cell_100	1.265	−0.639	−0.211
MS4A1	Cell_100	−0.314	−0.275	−0.341
CD8A	Cell_100	−0.307	0.307	0.501
